# Supplementary material for: Cajanolactone A from Cajanus cajan Promoted Osteoblast Differentiation in Human Bone Marrow Mesenchymal Stem Cells via Stimulating Wnt/LRP5/β-Catenin Signaling
Source: Molecules. 2019 Jan 12;24(2):271. doi: 10.3390/molecules24020271 (PMC6358999; doi:10.3390/molecules24020271)
Supplement: Supplementary file 1 [file molecules-24-00271-s001.pdf]

Supplementary materials of compound 1

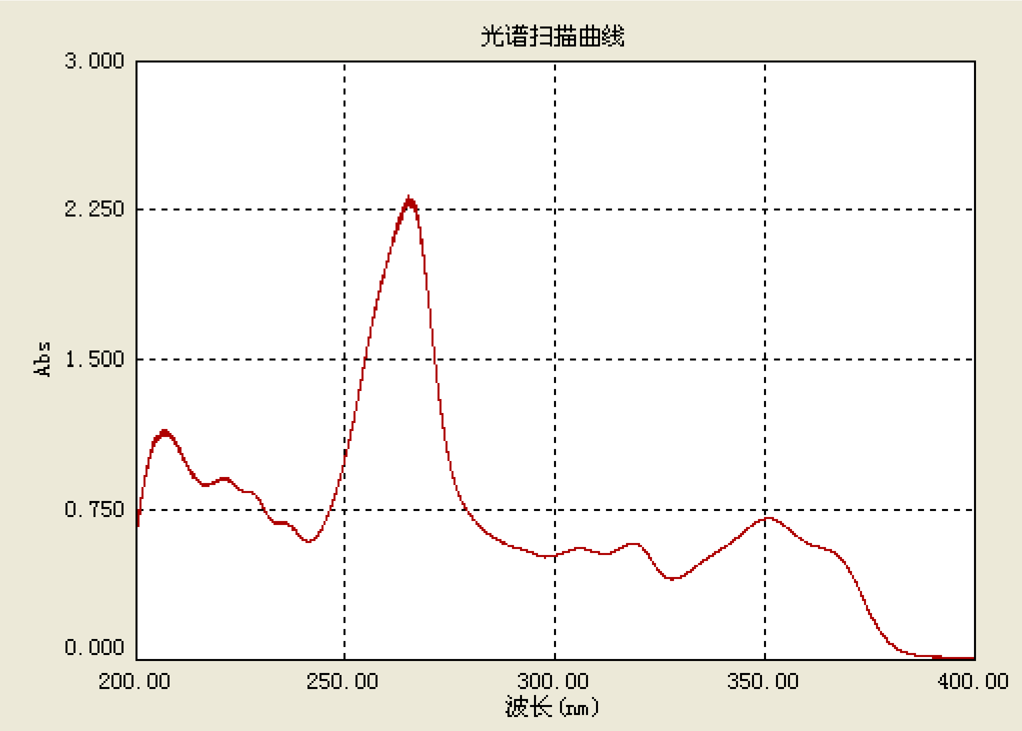

Figure 1. UV spectrum of 1

Concentration: 0.0099 mg/mL (methanol)

λ (nm)	Abs
206.8	1.153
265.3	2.326
351.0	0.708

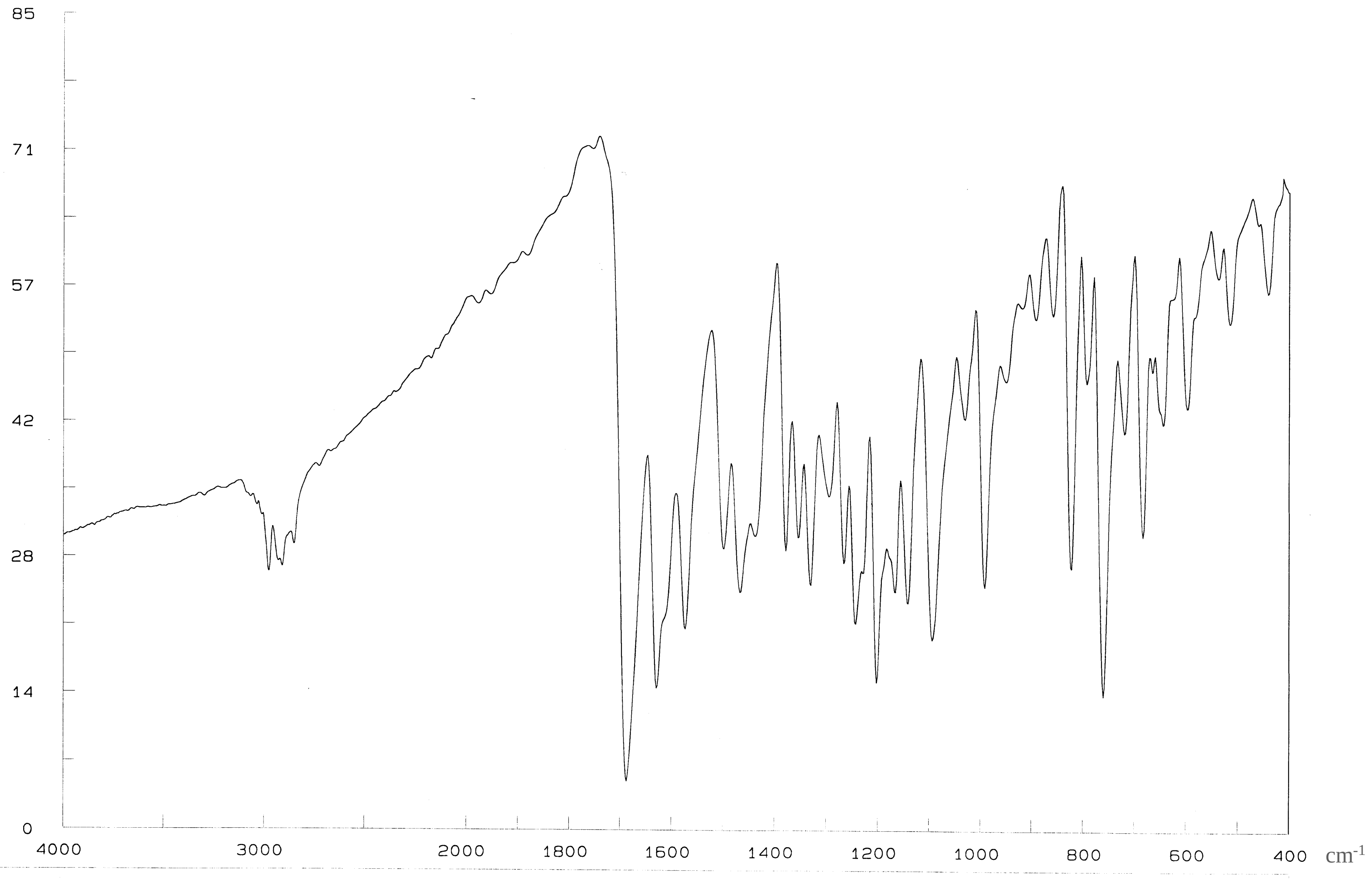

Figure 2. IR spectrum of 1

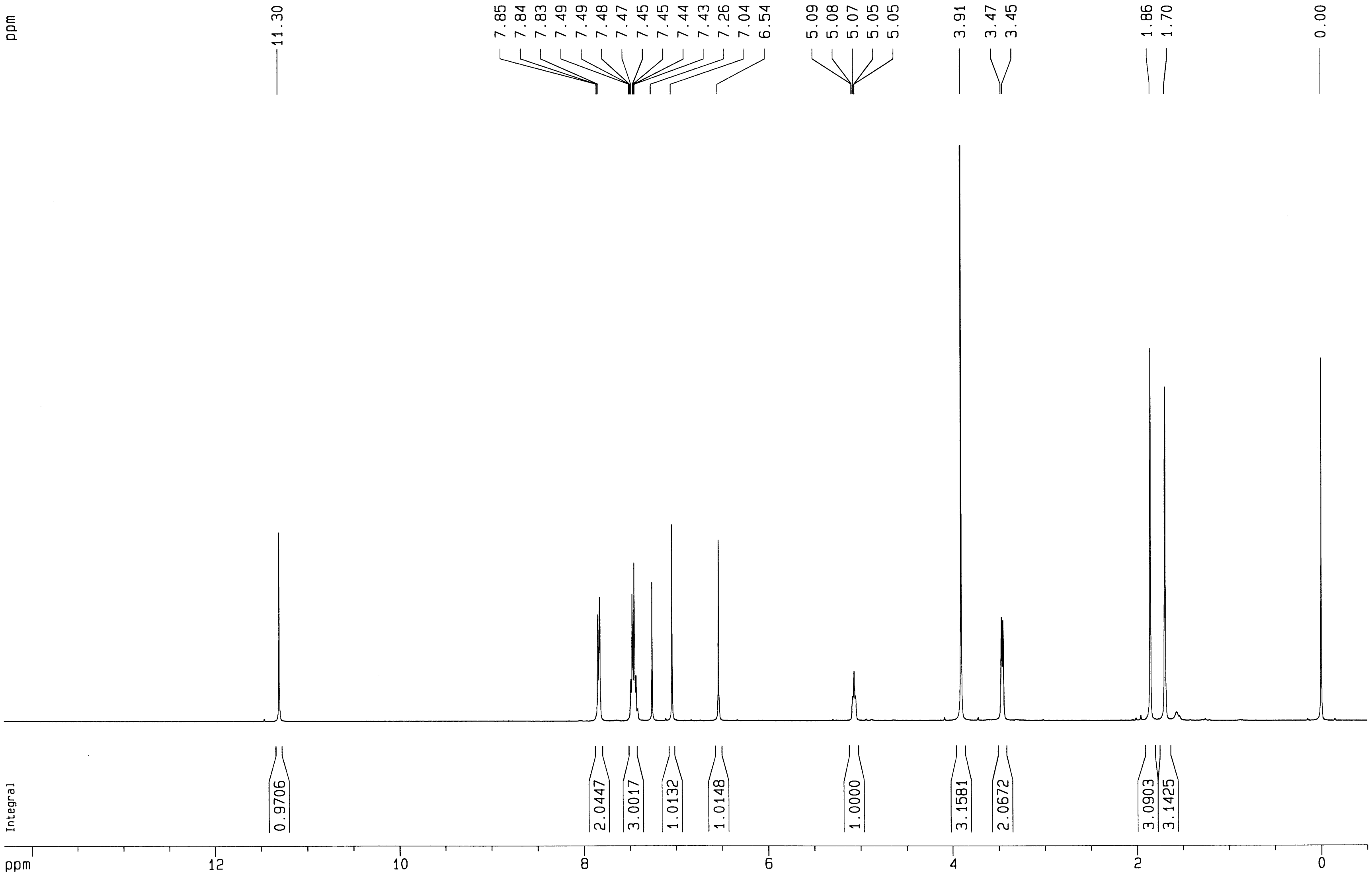

Figure 3. ^{1}H NMR spectrum of 1 (400 MHz, in $CDCl_3$)

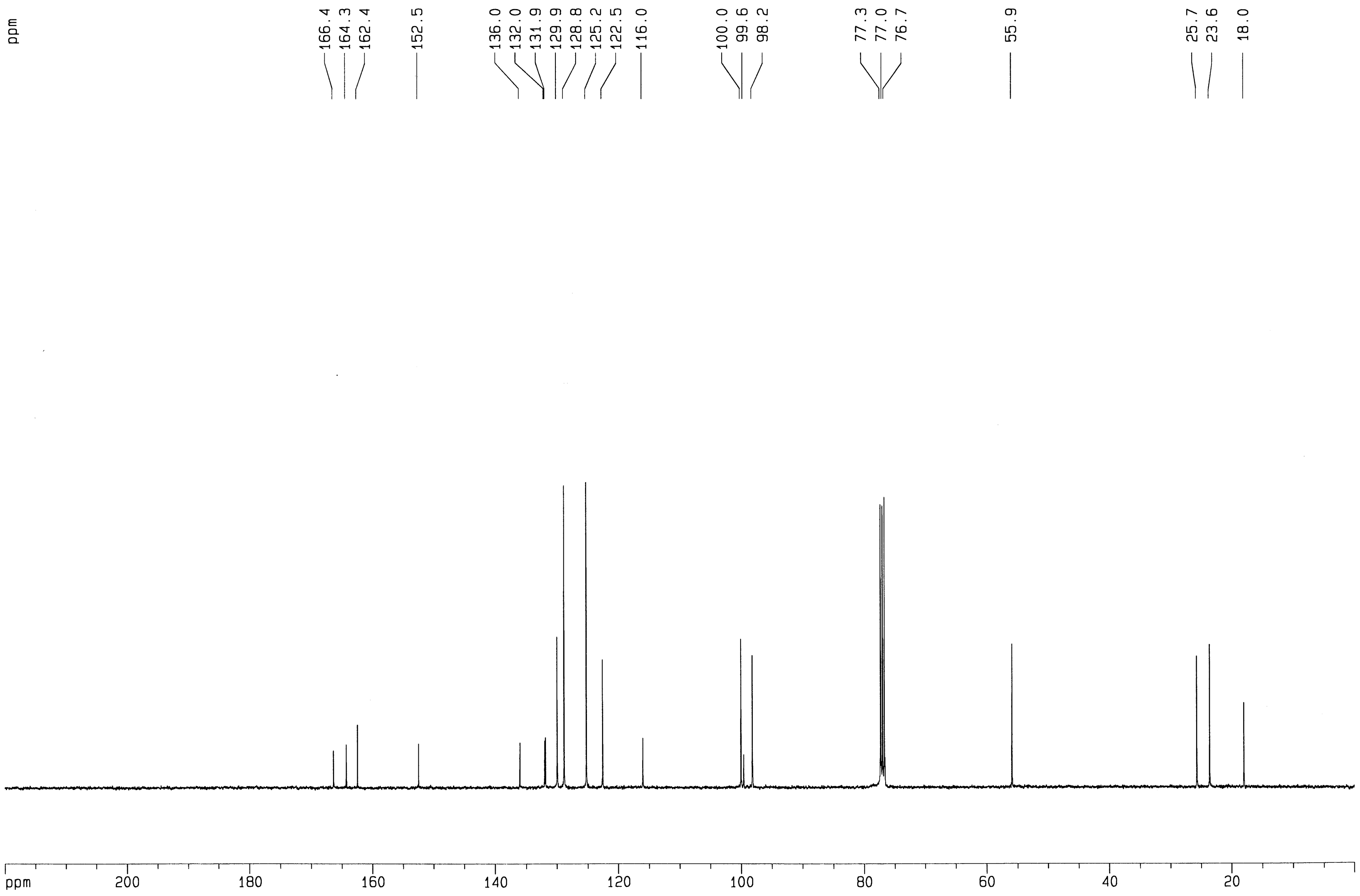

Figure 4. ^{13}**C NMR spectrum of 1 (100 MHz, in** $CDCl_3$**)**

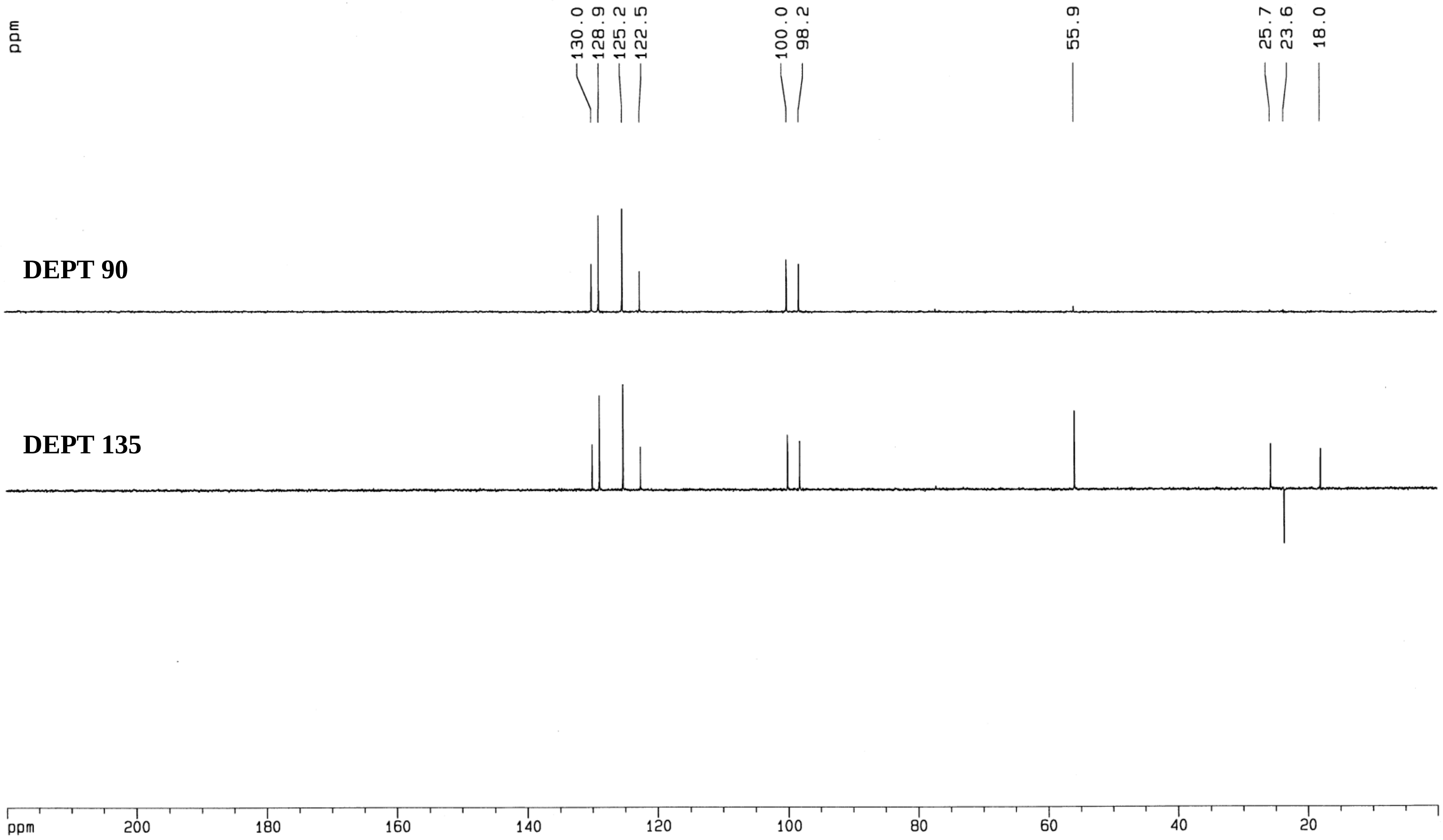

Figure 5. DEPT spectrum of 1 (100 MHz, in $CDCl_3$)

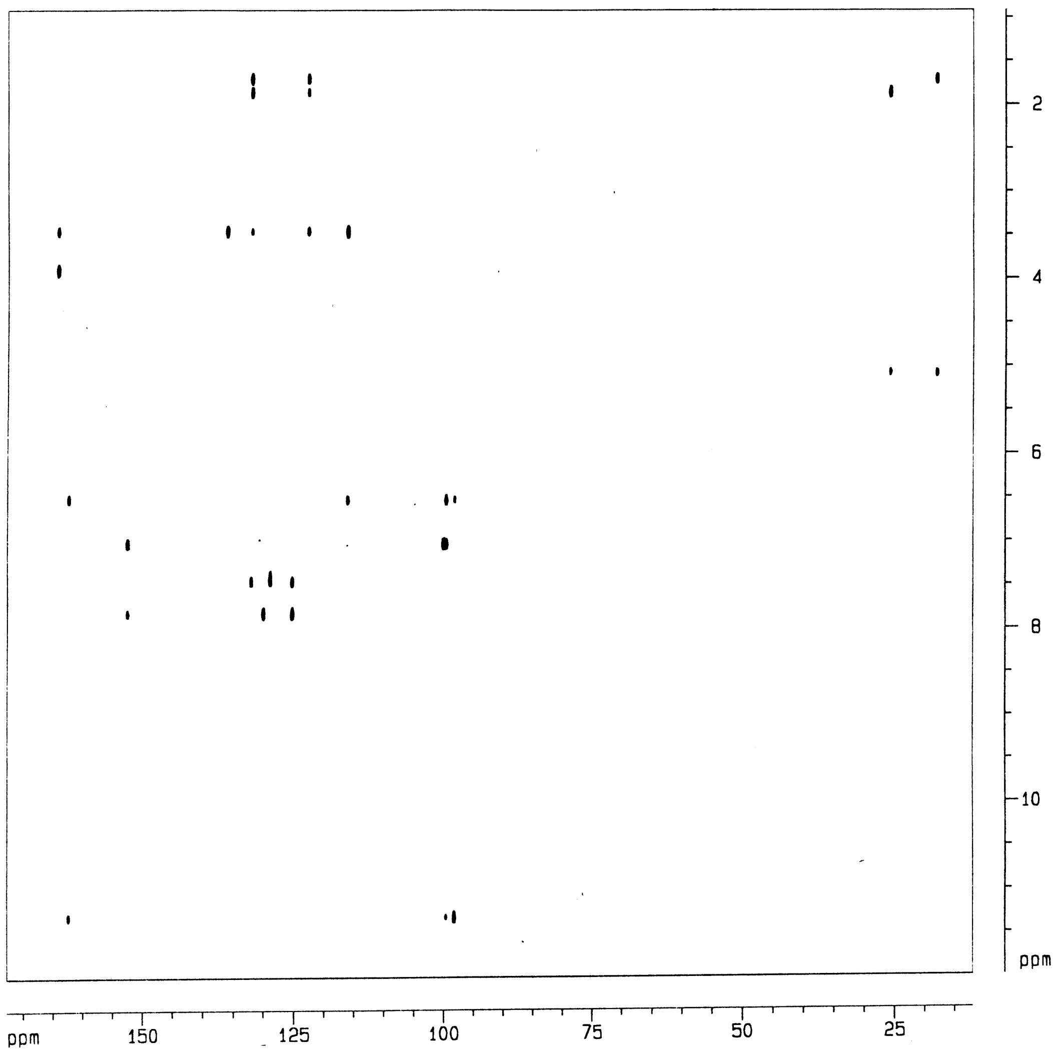

Figure 6. HMBC spectrum of 1 (in $CDCl_3$)

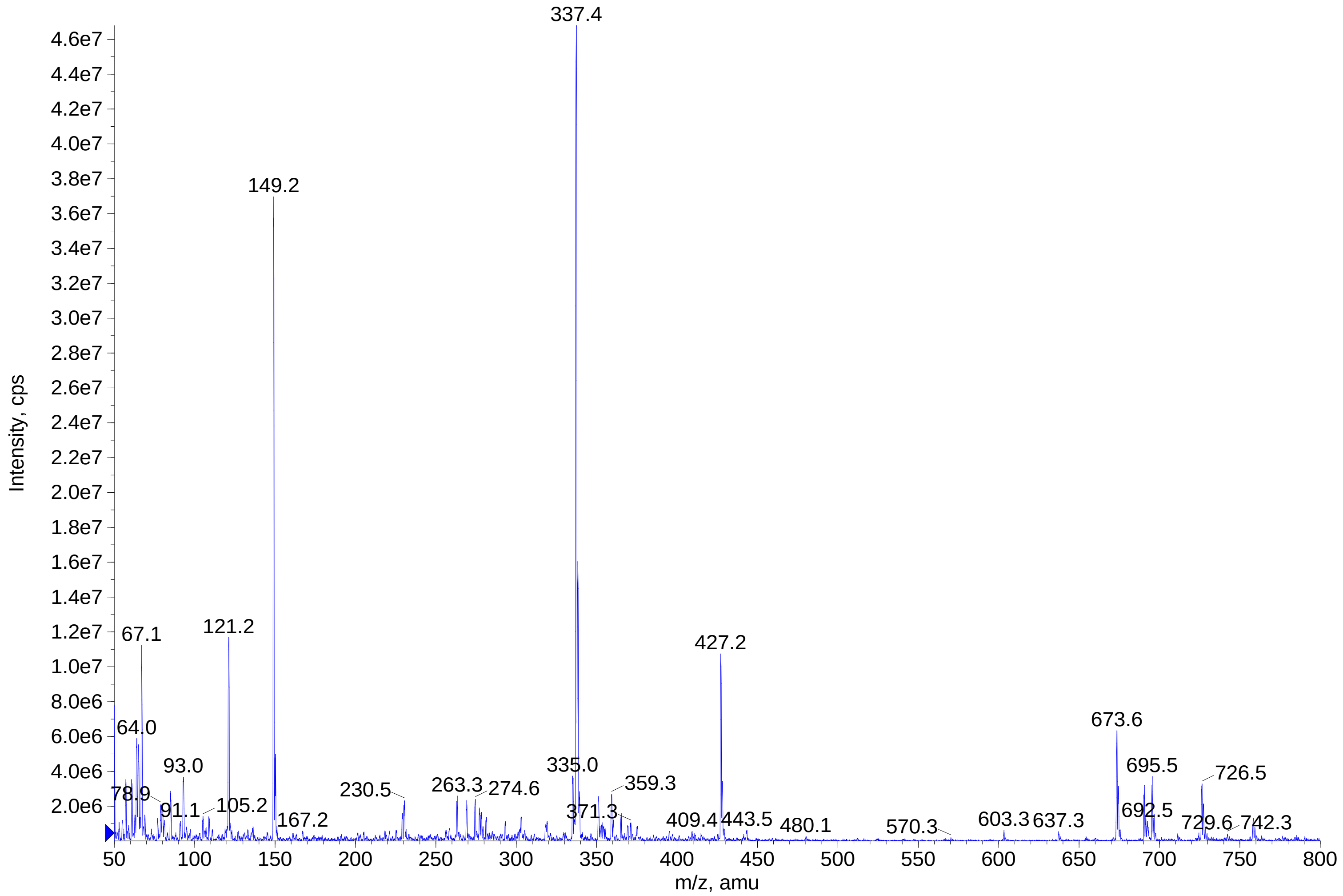

Figure 7. ESI MS spectrum of 1

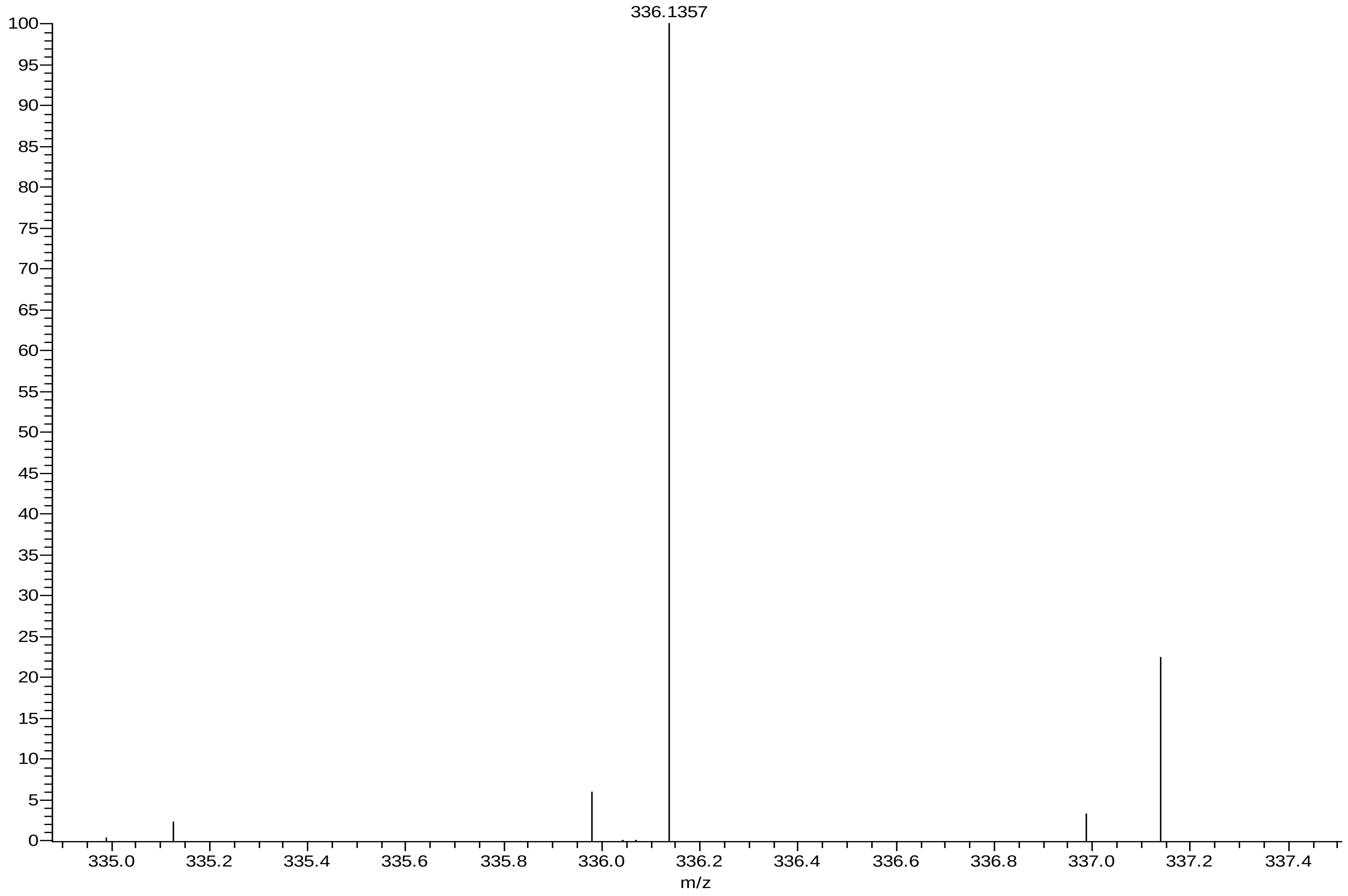

Figure 8. **HR EIMS spectrum of 1**

Assay of 1 by HPLC

Conditions and equipments

Chromatography was performed on an Agilent HPLC system with a VWD detector (Agilent Technologies Inc., USA). The separation was carried out on a Agilent HC-C18 (4.60 mm × 250 mm, 5 μm; Agilent Technologies Inc., USA) maintained at 25 ℃, with mobile phase of methanol – water (90 : 10, v:v) and detection wavelength of 265 nm. The flow rate was set at 1.0 mL/min. Sample solution was injected 20 μL.

Sample processing and result

Compound **1** was dissolved in methanol to afford test solution (0.1 mg/mL). The test solution was determined twice in parallel. The result was showed in Figure 9. Purity of 1 was determined as 99.0%.

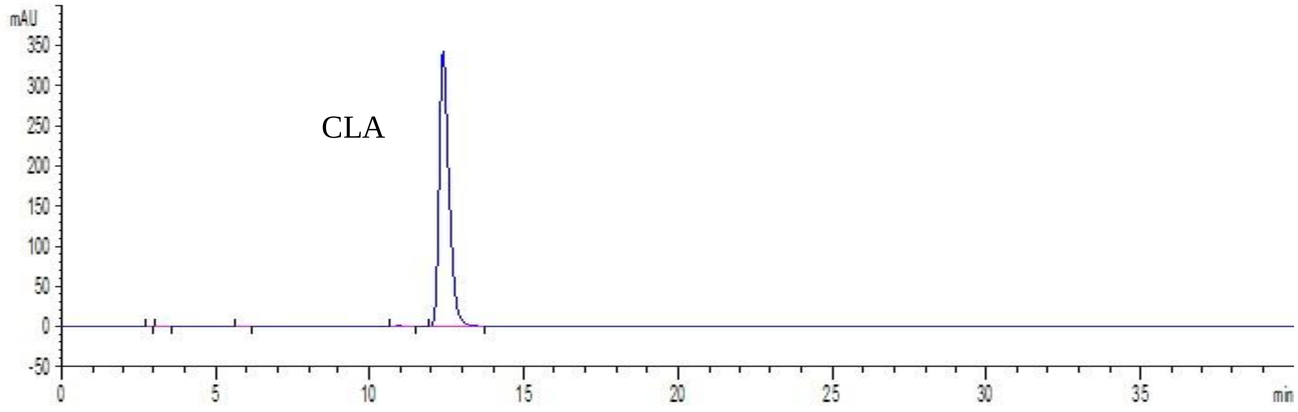

Figure 9. HPLC chromatogram of CLA
